# Exploring the impact of postponing core clerkships on future performance

**DOI:** 10.1080/10872981.2022.2114864

**Published:** 2022-09-04

**Authors:** Jeffrey B. Bird, Doreen M. Olvet, David Orner, Joanne M. Willey, Judith M Brenner

**Affiliations:** a Department of Science Education, Donald and Barbara Zucker School of Medicine at Hofstra/Northwell, Hempstead, NY, USA; b Office of Academic Affairs, Northwell Health, New Hyde Park, NY, USA

**Keywords:** Experiential learning, assessment, undergraduate medical education, clerkship

## Abstract

Despite the many clerkship models of medical education, all can be considered a form of experiential learning. Experiential learning is a complex pedagogical approach involving the development of cognitive skills in an environment with a unique culture with multiple stakeholders, which may impact learner motivation, confidence, and other noncognitive drivers of success. Students may delay the transition to the clerkship year for myriad reasons, and the intricate nature of experiential learning suggested this may impact student performance. This retrospective, observational study investigated the impact of clerkship postponement by measuring subsequent clerkship performance. Pre-clerkship and third-year clerkship performance were analyzed for three cohorts of students (classes of 2018, 2019, and 2020, N = 274) where students had the option to delay the start of their clerkship year. A mixed analysis of variance (ANOVA) and paired t-tests were conducted to compare academic performance over time among students who did and did not delay. Across three cohorts of students, 12% delayed the start of the clerkship year (N = 33). Regardless of prior academic performance, these students experienced a significant reduction in clerkship grades compared to their non-delaying peers. Delaying the start of the clerkship year may have negative durable effects on future academic performance. This information should be kept in mind for student advisement.

## Introduction

The clerkship model of medical education is broadly regarded as experiential learning [1]. While this may seem self-evident, the term ‘experiential learning’ does not describe a single pedagogical theory, but rather an amalgam of learning theories reflecting the complex learning environment and its diverse stakeholders [1]. Chief among these is situativity theory, which blends situated cognition and situated learning theories to posit that learning and knowledge grow out of experiences that include multiple participants and occur within an environment with a unique culture. Situativity theory predicts that learning and transfer of that learning to new circumstances comprises evolving interactions between participants and environment [2]. In the context of situativity theory, clerkships can be considered cognitive apprenticeships, whereby learning occurs while working within a community of practice with medical students engaging in legitimate peripheral participation, contributing to authentic patient care under the guidance of coaches, mentors, and near-peer teachers [2]. Clerkship experiences are designed to build knowledge and skills, but student confidence, motivation, sense of belonging, and burgeoning professional identity are also impacted [2]. Taken together, this framework illustrates the complexity of clerkship-based learning.

Given the complexity of experiential learning, it is unsurprising that performance in core clerkships lacks a universally agreed upon grading standard [3,4]. Instead, individual schools commonly utilize a variety of assessment modalities. Each student’s performance likely reflects both the cognitive and affective impact of experiential learning.

An individual student’s progression through the medical education curriculum may be interrupted by any number of personal or academic factors [5–7]. For several years at the Donald and Barbara Zucker School of Medicine (ZSOM), students had the option of postponing the start of their clerkships by six-weeks to gain additional time to prepare for the USMLE Step 1 exam. Students who used this option either were lower performing students who may have been in danger of failing the Step 1 or higher performing students who thought that the additional 6 weeks of preparation would increase their Step 1 score to a more competitive level. Given the intricate nature of clerkship learning, we hypothesized that delaying the start of the clerkship year would negatively impact student academic performance. To our knowledge, this is the first study to explore the relationship between postponing clerkships and future academic performance.

## Materials and methods

We performed a retrospective analysis of 33 students from the classes of 2018 (N = 10, 11%), 2019 (N = 13, 14%), and 2020 (N = 10, 11%) at one institution (ZSOM) who opted to postpone their first clerkship (delay group). Only students who opted to delay the clerkships for 6-weeks in order to study for Step 1 were included in this study. The remaining students in each of these classes were included in the no-delay group (Class of 2018, N = 78; Class of 2019, N = 81 and Class of 2020, N = 82). Pre-clerkship performance was calculated by combining scores of 21 assessments taken during the first and second years of medical school, including essay-based medical knowledge and laboratory exams as well as National Board of Medical Examiners (NBME) Customized Assessment Service exams. Scores for each assessment were converted to cohort specific z-scores and then averaged across all exams into a pre-clerkship z-score.

Third-year clerkship performance was calculated by converting clerkship grades into a z-score and then averaging all clerkship z-scores into an overall clerkship z-score. For each of the six core clerkships (Internal Medicine, Surgery, Psychiatry, Neurology, Obstetrics and Gynecology, and Pediatrics), the final grade was calculated with the following weighting: NBME Clinical Subject exams (30%), observed structured clinical skills exams (25%), and assessment rating forms completed by clinical faculty and residents (45%).

We performed a mixed analysis of variance (ANOVA) to detect potential interaction between the change in student performance over time (pre-clerkship vs. clerkship performance) and the choice to delay. *Post-hoc* paired samples t-tests were conducted to examine how performance from the pre-clerkship to the clerkship period changed in delayers versus non-delayers.

Lastly, students with a history of academic difficulty during the pre-clerkship years were analyzed independently from students lacking prior academic difficulty, defined as scoring more than one standard deviation below their class mean on at least two of their pre-clerkship final exams. A mixed ANOVA was conducted on each group independently to determine if student performance changed over time in both higher and lower performing students with delay as a between-subjects variable.

## Results

Of the 274 students in the classes of 2018, 2019, and 2020, 33 (12%) delayed their clerkships by six weeks. Among the delay group, eight students (24%) had little or no history of academic difficulty, and 25 students (76%) had a history of prior academic difficulty.

Results of the mixed ANOVA indicated a significant change in performance from pre-clerkship to clerkship grades (*F*(1,272) = 6.93, *p *< 0.01). Furthermore, there was a significant interaction between student performance and delaying (*F*(1,272) = 12.07, *p *< 0.001). *Post hoc* paired t-tests revealed that students who delayed had a significant reduction in their academic performance during their clerkship year (t(32) = 2.55, *p *= 0.02), while academic performance in students who did not delay remained the same over time (t(240) = 1.27, *p *= 0.21).

**Figure 1.**
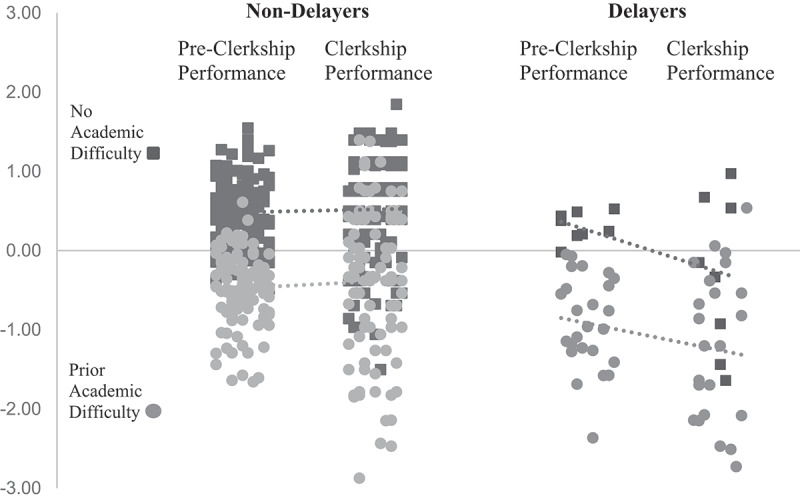
Effect of delaying clerkships on student performance in students with and without prior academic difficulty.

Among students with and without prior academic difficulty, mixed ANOVAs revealed a significant interaction between students’ performance and delaying for both those with (*F*(1,146) = 7.17, *p *< 0.01) and without any history of academic difficulty (*F*(1,124) = 6.11, *p *< 0.05), indicating that delaying clerkships negatively affected all students (Figure 1).

## Discussion

Delaying the start of the clerkship year is not uncommon. When making the decision to delay, students and faculty likely focus on the acute reason motivating their request for delay. The choice to delay may have been influenced by circumstances that could have impacted short-term focus without considering any potential long-term impact of the decision.

Our study demonstrates that for the majority of students, delaying the start of the clerkship experience was associated with lower overall clerkship grades regardless of previous academic performance. One might have expected these effects to be mitigated over time, yet we observed clerkship performance for delayers was lower than the non-delay cohort for the clerkship year as a whole. Although students who delayed were required to attend the same 2-week pre-clerkship bootcamp and orientation sessions as their non-delaying peers, starting their clerkship year late was associated with enduring deleterious effects. A possible explanation may be that delaying students felt ‘out of sync’ with their classmates. They are starting their first clerkship when the rest of their peers have already gained some familiarity with the expectations of their clerkship year. Although planned delays for students struggling in the pre-clerkship years have been found to prevent future academic failures when students are given an opportunity to spread their coursework over more time [8], students who delayed clerkships to study for Step 1 were not given the benefit of extending coursework and were expected to graduate on time. Students may have felt additional stress from a more condensed schedule, despite actual clerkship lengths not being reduced.

Although there is an overall downward trend from pre-clerkship to clerkship performance for students who delayed, not all had a decrease in performance in the clerkship year. This is not surprising given the complex nature of experiential learning where the interactions between a student and the learning environment can have a positive or negative effect on student learning. For the delaying students who did not see a negative impact on their performance, we hypothesize their first clerkship experience may have been bolstered by joining a particularly strong team or finding a mentor to help guide them.

Our study is not without limitations. We examined a relatively small cohort of students at a single institution. In addition, the sample size for students who choose to delay is small, especially those who delay with no previous academic difficulty. This may amplify the impact of outliers. As an observational study, we did not address why most students’ clerkship performance was lower but suggest that it may reflect the complex nature of experiential learning [1,2]. Indeed, future studies that seek to define the reasons the delay of the clerkship year had a broad and durable impact should include potential psychosocial factors such as learner self-efficacy [9] and self-determination [10].

When faced with student requests to delay their clerkship year, medical school leadership should include the long-term impact on grades in their advising. Although other outside factors need to be considered when making such an important decision, these should be carefully weighed against the potential impact on students’ academic future performance.

## Data Availability

Deidentified data collected from the Donald and Barbara Zucker School of Medicine at Hofstra/Northwell are available from the corresponding author upon reasonable request.
